# Mitigation of Lost Circulation in Oil-Based Drilling Fluids Using Oil Absorbent Polymers

**DOI:** 10.3390/ma11102020

**Published:** 2018-10-18

**Authors:** Hanyi Zhong, Guangcheng Shen, Peng Yang, Zhengsong Qiu, Junbin Jin, Xiaodong Xing

**Affiliations:** 1 School of Petroleum Engineering, China University of Petroleum (East China), Qingdao 266580, China; m18754272192@163.com (G.S.); qiuzs@upc.edu.cn (Z.Q.); 2 GWDC Engineering Research Institute, Panjin 124010, China; yangpeng00625@163.com; 3 SINOPEC Research Institute of Petroleum Engineering, Beijing 100101, China; jinjb.sripe@sinopec.com; 4 China Petroleum Engineering & Construction CORP, North China Company, Renqiu 062552, China; xingxiaodong@cpeccnc.com

**Keywords:** lost circulation, oil-based drilling fluid, oil absorbent polymer, fracture sealing, combination

## Abstract

In order to mitigate the loss circulation of oil-based drilling fluids (OBDFs), an oil-absorbent polymer (OAP) composed by methylmethacrylate (MMA), butyl acrylate (BA), and hexadecyl methacrylate (HMA) was synthesized by suspension polymerization and characterized by Fourier transform infrared spectroscopy (FT-IR), thermogravimetric analysis (TGA) and scanning electronic microscopy (SEM). The oil-absorptive capacity of OAP under different solvents was measured as the function of temperature and time. The effect of the OAP on the rheological and filtration properties of OBDFs was initially evaluated, and then the sealing property of OAP particles as lost circulation materials (LCMs) was examined by a high-temperature and high-pressure (HTHP) filtration test, a sand bed filtration test, a permeable plugging test, and a fracture sealing testing. The test results indicated that the addition of OAP had relatively little influence on the rheological properties of OBDF at content lower than 1.5 *w*/*v* % but increased the fluid viscosity remarkably at content higher than 3 *w*/*v* %. It could reduce the HTHP filtration and improve the sealing capacity of OBDF significantly. In the sealing treatment, after addition into the OBDF, the OAP particles could absorb oil accompanied with volume enlargement, which led to the increase of the fluid viscosity and slowing down of the fluid loss speed. The swelled and deformable OAP particles could be squeezed into the micro-fractures with self-adoption and seal the loss channel. More important, fluid loss was dramatically reduced when OAP particles were combined with other conventional LCMs by a synergistic effect.

## 1. Introduction

In oil and gas drilling engineering, one of the frequently encountered problems is lost circulation, which is defined as the undesirable partial or complete loss of drilling fluid into formation voids during drilling, circulation, running casing, or cementing operations [1,2]. Once the total pressure exerted against the formation exceeds the formation breakdown pressure, lost circulation may be encountered at any depth. According to the statistics, the occurrence of lost circulation is present in approximately 20 to 25% of wells drilled around the world during drilling [3], and results in several troublesome problems such as excessive mud losses, non-productive time, stuck pipe, well kick, well blow-out and even abandonment of the wells [4,5,6,7]. Moreover, it has also been blamed for minimized production because the loss of fluid into a formation plugs the production zones and leads to decreased productivity [8,9]. More than 2 billion USD is spent to combat and mitigate this problem each year [10,11]. Lost circulation poses great challenges for the industry.

Lost circulation is generally observed in four kinds of formation including natural or induced fractured formations, vugular or cavernous formations, highly permeable formations, and unconsolidated formations [3]. Although the forms of loss like filtration loss, matrix seepage, and vugular loss may be of concern, losses through fracture propagation is the primary type and accounts for over 90% of the operator lost returns expenditures [7].

Over the past years, much effort has been made in an attempt to mitigate or stop lost circulation. A wide variety of lost circulation materials (LCMs) have been employed to form dense and integrated fracture sealing barriers [12]. The list of materials commercially available as LCMs is impressive [13,14]. Conventionally, these materials can be classified as fibrous, flaky, granular type, and mixtures of these [15]. For instance, particulate materials such as calcium carbonate and graphites, fibrous materials such as wood fiber, mineral fiber, and glass fiber; flake materials such as cellophane, mica and vermiculite; and granular materials such as perilite, nut shells and ground tires are widely used [16]. Meanwhile, other types of materials including cement, chemically activated cross-linked pilles, cross-linked cement, deformable-viscous-cohesive systems, nano-composite gel, gunk squeezes [17], polyurethane grouting, crosslinked gel, viscoelastic surfactant, nano-particles [18], reticulated foam [19], and shape memory polymers [20] have also attracted attention. These materials are generally added either to the drilling fluid or separately in the form of a sweep or a treating pill [21].

Because of the inherent advantages of high temperature stability, excellent lubricity, high wellbore stability, and tolerance to pollution and so on, invert emulsion drilling fluids (including oil-based drilling fluid (OBDF) and synthetic-based drilling fluid (SBDF)) are generally preferred when there are drilling demanding formations and complex conditions such as deep formation drilling, deep water drilling and gypsum and so on in comparison with water-based drilling fluids [22,23,24]. However, one potential drawback to the use of these fluids is the high cost associated with lost circulation [12]. On the one hand, the strong dependency of density on both temperature and pressure makes the invert-emulsion drilling fluid more compressible than the water-based drilling fluid, which subsequently results in a narrower drilling fluid density margin and easy occurrence of lost circulation [25,26]. On the other hand, for the pressure required to initiate hydraulic fracturing of the formation, there is no difference between water-based drilling fluid and invert emulsion drilling fluid; however, after the fractures are formed, there is a noticeable difference [9,27,28]. For water-based drilling fluid, a higher spurt fluid loss causes the almost instantaneous formation of a filter cake, and is then followed by a higher filtration loss, which results in the formation of thicker filter cakes, shielding the fracture tip from the maximum wellbore pressure and allowing bridges to develop that prevent further fracture propagation [26]. Meanwhile, the presence of filter cake can heal the fractures and lead to higher fracture re-opening pressures. Nevertheless, for invert emulsion drilling fluid, because of almost no dispersion of drilling cuttings, the filter cake is primarily composed of emulsion droplets and very thin and impermeable. Meanwhile, after wetting by the invert emulsion drilling fluid, the friction between LCMs and fracture plane decreases and cause the LCMs relatively difficult to bridging and forming effective sealing, which facilitate movement along these weak planes [29,30]. Therefore, once the fracture is initiated, a much smaller pressure is needed to propagate the fracture compared to water-based drilling fluid. This allows changes in wellbore pressures to be transmitted to the formation more readily, and further propagates the fracture [9,31]. Furthermore, although a variety of materials have been used as LCMs, materials specially designed to combat lost circulation in invert-emulsion drilling fluids are relatively fewer than that for water-based drilling fluids [25]. For solid particles, oil-wetting chemicals must typically be added to ensure the oil wet property when drilling with an OBDF [32]. Thereby, when using invert-emulsion drilling fluids, very few lost circulation remedies have been successful [33,34].

Water-absorbent resin is a kind of polymer with cross-linking structures which render the resin water-insoluble and capable of absorbing water of several to hundreds of times of its own weight. By deforming and being squeezed into the loss fractures with flexibility, the polymers can absorb water and fill in the fractures or pores. Due to the adsorption on the rock surface, it is easy for the polymers to stay in the loss channels and form a strong and pliable plug [35]. Because of the above advantages, water absorbent resin has been widely used in water-based drilling fluid as an effective LCM [36,37]. Behaving like water-absorbent resins, oil-absorbent resins having the cross-linked, three-dimensional, and hydrophobic networks that do not dissolve in oil are mainly used for absorbing oil in environmental pollution treatment [38]. Therefore, considering the similar properties between oil-absorbent resin and water-absorbent resin, the object of the current study is to probe the feasibility of oil absorbent resin in mitigating the loss of OBDFs.

## 2. Materials and Methods 

### 2.1. Materials

Monomers including methylmethacrylate (MMA) (99%), butyl acrylate (BA) (98%), and hexadecyl methacrylate (HMA), cross-linking reagent *N*,*N*’-Methylenebis (acrylamide) (MBA) (99%) and initiator benzoyl peroxide (BPO) were purchased from Shanghai Aladdin Biochemical Technology Co. Ltd. (Shanghai, China) with analytical purity. Polyvinyl alcohol (PVA) using as dispersant agent, ethyl acetate (EAC) as porogen, and ethanol were bought from Sinopharm Chemical Reagent Co., Ltd. (Beijing, China) with analytical purity. 

The low-toxicity mineral oil of No. 3 white oil used as the base oil was purchased from Shandong Taichang Petrochemical Technology Co., Ltd. (Qingdao, China). The organic clay with commercial name VG-Plus was provided by M-I Swaco Company of Schlumberger. Primary emulsifier BZ-OPE (amidoamine type) and secondary emulsifier BZ-OSE (fatty acid type) were provided by China National Petroleum Corporation (Tianjin, China). Rheological modifier SD-RM prepared from a reaction between polyacid and polyethene polyamine was provided by Shandong Shida Chuangxin Technology Co., Ltd. (Dongying, China). Barite used as weighting material was purchased from An County Huaxi mineral powder Co., Ltd. (Mianyang, China). Two types of conventional fluid loss additives including asphaltic additive and modified lignite used in OBDFs were provided by China National Petroleum Corporation and Shandong Shida Chuangxin Technology Co., Ltd. (Dongying, China), respectively. Lime (CaO, pH enhancer) and CaCl_2_ were bought from Sinopharm Chemical Reagent Co., Ltd. (Beijing, China). Calcium chloride was purchased from Sinopharm Chemical Reagent Co., Ltd. with analytical purity. The sized calcium carbonate (SCC) particles were provided by Jingmen Shun Zhan calcium Industry Co., Ltd. (Jingmen, China). The rubber (RUB) particles were obtained from Dujiangyan Huayi Rubber Co., Ltd. (Dujiangyan, China). The fibers (FIB) using as LCM were bought from Changzhou Tianyi engineering fiber Co., Ltd. (Changzhou, China). All the reagents were used as received without further purification.

### 2.2. Synthesis and Characterization of Oil-Absorbent Polymer (OAP)

The reaction was performed in 500 mL, four-necks, round-bottom flasks equipped with a mechanical stirrer and heating device. Initially, 240 mL of PVA (2 g) solution was added into the flask and stirred for 30 min with water bath heated to 60 °C for facilitation of dissolution. The system was charged with nitrogen gas and then sealed under nitrogen. Then, a mixture containing monomers of MMA (6 g), BA (16 g) and HMA (18 g), cross-linker MBA (0.3 g), initiator BPO (0.4 g) and porogen EAC (5 g) was added into the reactor within 10 min. The polymerization reaction was performed at 80 °C for 6 h under a stirring rate of 600 rpm. After reaction termination, the products were washed with absolute ethanol several times and then washed with hot deionized water (60–70 °C) several times. After washing, the sample was dried in vacuum drying oven (Qingdao Haitongda Special Instrument Co., Ltd., Qingdao, China) at 55 °C for 24 h and finally the product of oil-absorbent polymer (OAP) was obtained as small beads. The particle size can be controlled by adjusting the stirring rate and the ratio of reaction monomers.

The Fourier transform infrared (FT-IR) spectra were recorded by a Nicolet 6700 FT-IR spectrometer (Thermo Fisher Nicolet Corporation, Waltham, MA, USA), scanning from 4000 to 400 cm^−1^, with 4 cm^−1^ resolution in transmission. A TGA/DSC 1/1600 HT thermal analyzer from Mettler Toledo (Zurich, Switzerland) was used for thermogravimetric analysis (TGA) with a heating program from room temperature to 1000 °C at a heating rate of 10 K min^−1^ in nitrogen flow of 50 mL min^−1^. The morphological features of the OAP were inspected with FEI Quanta FEG 250 field-emission scanning electron microscope (SEM, Hillsboro, OR, USA). The oil-adsorption capacity was conducted with the weighting method [39]. A quantity of 1 g of dried OAP samples was put into a filter bag and immersed in oil at a certain temperature. After a period of oil absorption, the filter bag with the sample was lifted from the oil and drained for 1 min. Then the sample was immediately taken out, weighed and recorded. The oil absorbency was calculated as follows:

(1) $$\;R=\frac{\left(W-1\right)}{1}\;$$

where, R is the oil absorbency at a certain testing time, g/g; *W* is the weight of OAP after oil adsorption for a certain testing time, g.

### 2.3. Preparation of Oil-Based Drilling Fluids (OBDFs)

The mineral oil-based drilling fluids were prepared according to the experimental methods recommended in API RP 13B-2 [40]. The drilling fluid formula with oil to water ratio (OWR) of 90:10 is listed in Table 1. When the OWR of the fluid changed, the concentration of primary emulsifier and assistant emulsifier would be adjusted correspondingly to ensure the emulsion stability. The fluids were hot rolled in a rolling oven (Qingdao Haitongda Special Instrument Co., Ltd., Qingdao, China) at a certain desired temperature for 16 h. After the dynamic aging, the fluids were cooled down to room temperature and agitated for 10 min at 10,000 rpm before it was analyzed. 

### 2.4. Rheological Properties and Electrical Stability Measurement 

The rheological properties of the fluids were carried out at 50 °C according to the standard American Petroleum Institute Recommended Practice (API RP) 13B-2. The rheological parameters including apparent viscosity (AV), plastic viscosity (PV), yield point (YP), and gel strength of the OBDFs were measured using a model ZNN-D6 six-speed rotating viscometer (Qingdao Haitongda Special Instrument Co., Ltd., Qingdao, China). The AV, PV and YP were calculated from 300 and 600 rpm readings by the following equations:

Apparent viscosity (AV) = Φ600/2 (mPa·s)(2)

Plastic viscosity (PV) = Φ600 − Φ300 (mPa·s)(3)

Yield point (YP) = 0.48(Φ300 − PV) (Pa)(4)

The Gel_in_ and Gel_10min_ were recorded as the maximum dial reading at a fixed rate of 3 r/min after undisturbed for 10 s and 10 min, respectively [41]. The electrical stability (ES) of the OBDFs was measured using the electrical stability tester (Qingdao Shande Petroleum Apparatus Co., Ltd., Qingdao, China).

### 2.5. Filtration Properties Measurement 

Different filter presses are used to determine the filtration property of drilling fluids. The API filtrate volume of OBDFs before and after hot rolling was tested by a ZNZ-D3-type medium-pressure filtration apparatus (Qingdao Haitongda Special Instrument Co., Ltd., Qingdao, China). The volume of filtration was collected through filter paper as filtration medium under a fixed pressure of 0.7 MPa for 30 min as recommended with API standard.

**Figure 1:**
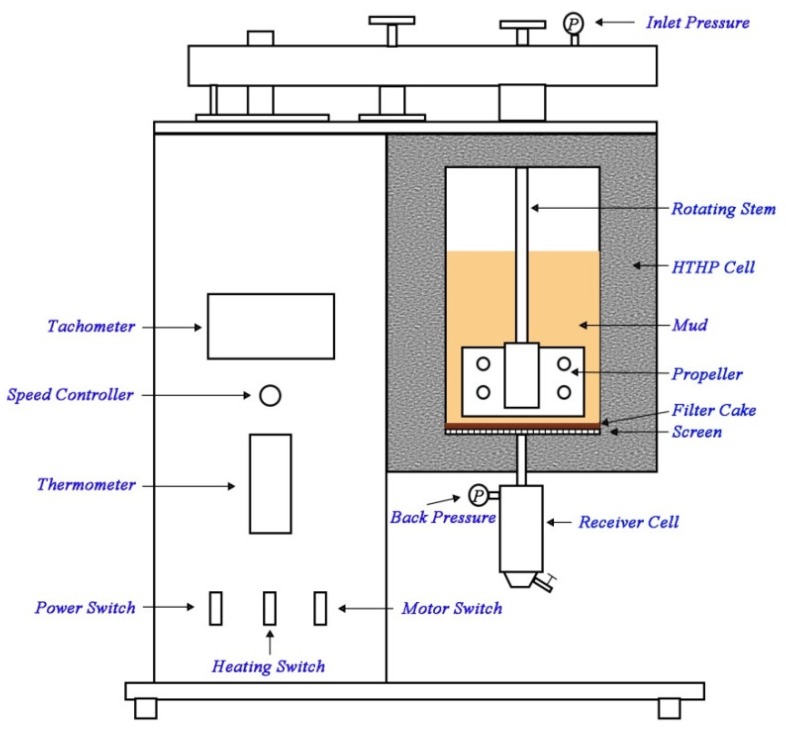
Scheme of high-temperature and high-pressure (HTHP) dynamic filtration cell.

In most situations, drilling fluid filtrate into the formation in the drilling is a dynamic process; therefore, the high-temperature and high-pressure (HTHP) dynamic fluid loss was measured with a HTHP dynamic filter press (Figure 1) (Qingdao Haitongda Special Instruments Co., Ltd.) at a stirring speed of 100 rpm. The tests were run for 30 min at a differential pressure of 3.5 MPa and 150 °C. The filtrate was collected with filter paper as filter medium.

When the circulation of drilling fluid stops because of accidents or downhole operations, a static filtration occurs. To simulate the static filtration behavior of drilling fluid, the HTHP static fluid loss was conducted via the HTHP filter apparatus (GGS71-B, Qingdao Haitongda Special Instruments Co., Ltd.) under the condition of a certain temperature and 3.5 MPa pressure difference for a period of 30 min. The volume of filtration and the thickness of the filter cake were recorded.

### 2.6. Properties of Sealing

To evaluate the sealing performance of OAP, four distinctive tests including HTHP filtration (including both static and dynamic filtration) using API filter paper as the filtration medium to simulate the permeable formation [42]; a permeability plugging test using ceramic disk as filtration medium to determine the ability of particles in the drilling fluid to bridge pores [43]; a sand bed filtration test using a sand bed as filtration medium to simulate an unconsolidated formation; and a fracture sealing test using wedged and slotted stainless steel as filtration medium to simulate a fractured formation were carried out [44,45].

The test procedure of the sand bed filtration test and permeability plugging test refer to Zhong et al. [46]. The sand particles used for sand bed filtration test have the particle size ranging from 380 µm to 830 µm. The sand disk using for permeability plugging test was calibrated to 10 D. 

**Figure 2:**
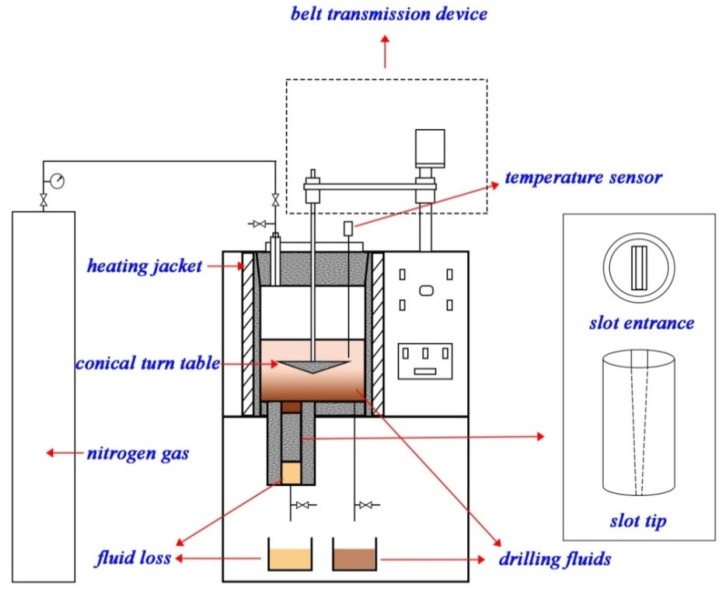
Fracture pressure bearing capacity test apparatus.

**Figure 3:**
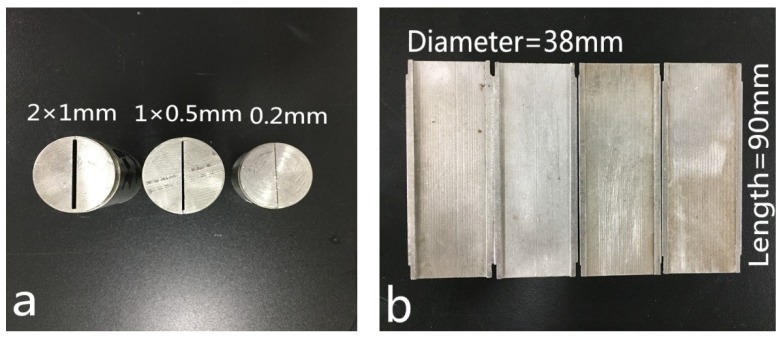
Tapered slots with various fracture sizes for fracture sealing testing. Note: (**a**) Size dimensions; (**b**) fracture profile.

In order to evaluate the fracture plugging capacity of LCMs, a fracture sealing testing apparatus (Figure 2, Instrument Factory of Petroleum University, Dongying, China) with tapered and slotted stainless steel discs (Figure 3) that simulate natural/induced fractures was adopted. Firstly, tapered slots were placed before the output valve. Then, fluids containing LCMs were forced to flow at a constant stirring rate of 100 rpm through the discs with a gradual increase of pressure. The applied pressure was initially set to be 1.0 MPa and kept stable for 10 min. If the LCMs effectively sealed the fracture and the pressure declined within 5%, the pressure was continuously increased and the volume of fluid loss was recorded. Once a continuous leakage of fluid occurred, it was asserted that the LCMs had reached the maximum pressure bearing capacity and the test was ceased. Treatments with lower fluid loss values correspond to effectiveness of mitigating the loss. However, because of the limitation that the maximum applied pressure is 8.0 MPa for the equipment, the maximum pressure at which the formed seal breaks and fluid loss resumes was not measured. 

## 3. Results

### 3.1. Characterization of OAP

#### 3.1.1. Fourier Transform Infrared (FT-IR) Spectra

**Figure 4:**
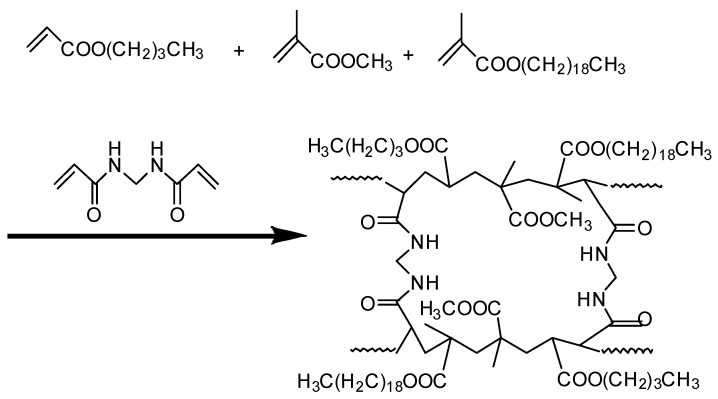
Possible chemical reaction mechanism for oil-absorbent polymer (OAP).

**Figure 5:**
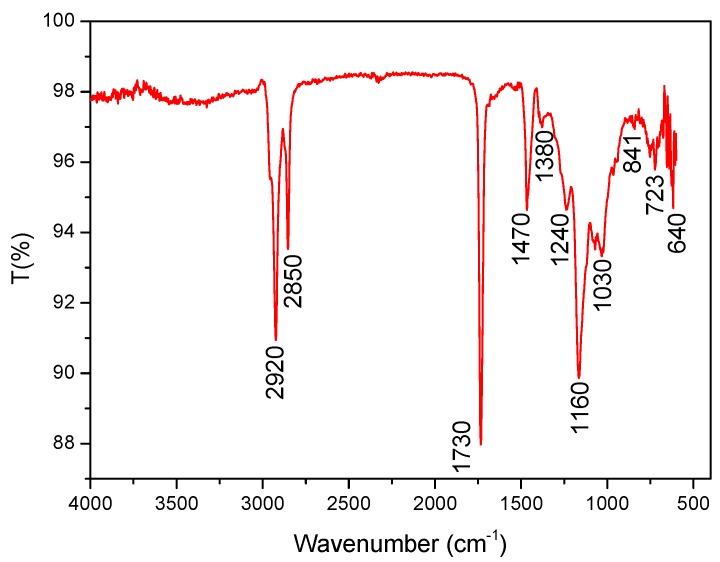
Fourier transform infrared (FT-IR) spectrum of oil-absorbent polymer (OAP).

A possible chemical reaction mechanism for OAP is depicted in Figure 4. The FT-IR spectrum of the copolymer prepared by suspension polymerization is shown in Figure 5. The characteristic absorption bands that appeared at 2920 and 2850 cm^−1^ were assigned to the asymmetric and symmetric stretching vibrations of C–H, respectively. The bands at 1730 cm^−1^ were attributed to C=O stretching vibration. The bands at 1470 and 1380 cm^−1^ corresponded to the C–H asymmetric and symmetric bending vibration. The bands at 1240 and 1160 cm^−1^ were the C–O–C asymmetric stretching vibration and symmetric stretching vibration, respectively. The band at 1030 cm^−1^ was assigned to the C–N stretching vibration. The bands at 723 and 640 cm^−1^ were the out of plane bending vibration of N–H (amide V) and C=O (amide VI), respectively. The disappearance of C=C bands usually at 1620–1680 cm^−1^ indicated the thorough reaction of the monomers.

#### 3.1.2. Thermogravimetric Analysis (TGA)

**Figure 6:**
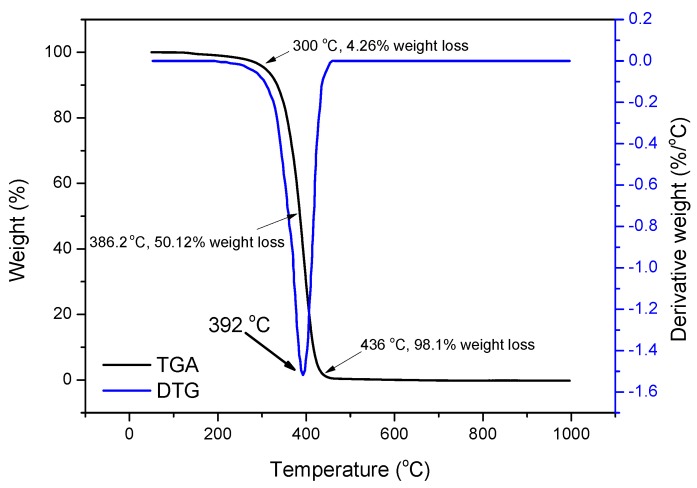
Thermogravimetric analysis (TGA) and Derivative thermogravimetric analysis (DTG ) curves of OAP.

The thermal stability is of vital importance for OAP because it has to experience downhole high temperature conditions. Figure 6 presents the resolution of weight percent of OAP sample with the change of temperature and the first derivative thermogravimetric curve. It could be seen that before 100 °C little weight loss was observed. The copolymer began to degrade rapidly when the temperature reached 300 °C. The sample weight loss is about 50.12% at 386 °C. The maximum pyrolysis rate occurred at about 392 °C. When the temperature reached 436 °C, the sample came to the total pyrolysis. The results indicated that OAP had a good thermal stability and could be used in high temperature environments.

#### 3.1.3. Scanning Electron Microscopy (SEM)

**Figure 7:**
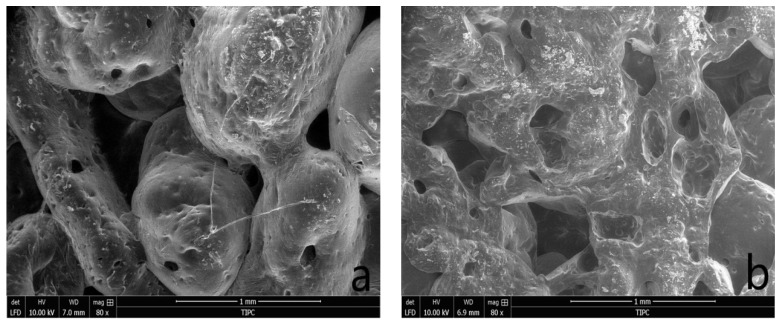
Scanning electron microscope (SEM) microphotographs of OAP. Note: (**a**) Surface; (**b**) cross section.

In order to observe the microstructure of OAP, SEM was used to inspect the cross section and surface morphologies of OAP samples, as depicted in Figure 7. From Figure 7a, aggregated micrometer-sized spheres were clearly observed. It is worth mentioning that many small pores were randomly distributed in the samples. There pores support the favorable space in the polymer network. The three-dimensional network structure and micro-pores with proper size and quantity was beneficial for the oil molecules to enter into the internal space; however, it is not easy for the oil molecules to exude from the three-dimensional crosslinked resin, like a sponge [47].

### 3.2. Oil-Adsorptive Capacity 

**Figure 8:**
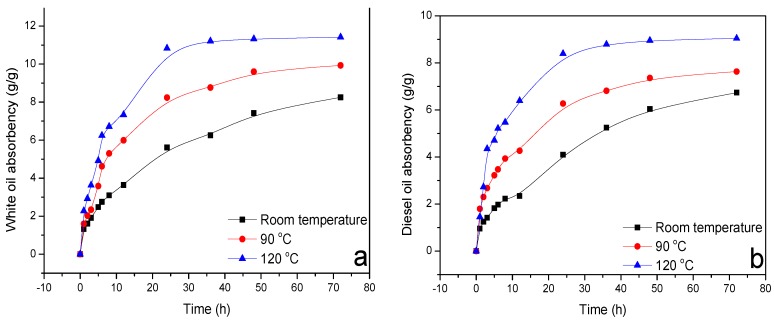
Evolution of oil adsorptive rate with time under various temperatures. Note: (**a**) white oil; (**b**) diesel oil.

The evolution of oil adsorptive rate which is calculated with Equation (1) with time is depicted in Figure 8. As can be seen from Figure 8, for both No. 3 white oil and diesel oil, a quick adsorption rate was observed for the initial testing interval of 20 h, followed by a slightly increased adsorptive rate, and it finally reached saturated adsorption. After testing for 72 h, the white oil absorbency was 8.3, 9.9, 11.4 g/g for testing temperature of room temperature, 90 °C and 120 °C, and the diesel oil absorbency was 6.7, 7.6, 9.0 g/g accordingly. Based on the field experience, the saturation adsorption time of water absorbent resin higher than 5 h would be sufficient for the resin to be injected into the downhole and fulfil its role [48], therefore for OAP, it would not become adsorption saturation before reaching the desired thief zone, which is advantageous for downhole application. OAP particles would still absorb oil when entering the loss fractures and prevent fluid loss. Meanwhile, the oil-adsorptive rate increased with the increasing testing temperatures, implying that OAP would adsorb a larger amount of oil in the downhole environment. Moreover, the saturation adsorptive amount of white oil was higher than that of diesel oil, demonstrating that OAP may be more effective in white oil-based drilling fluid. 

**Figure 9:**
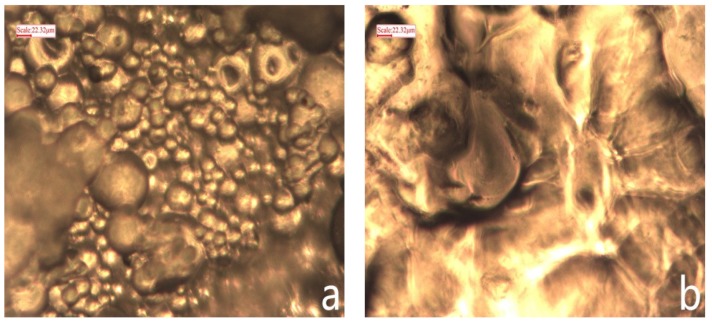
Microphotogram of OAP particles before (**a**) and after (**b**) No. 3 white oil adsorption.

Optical microscope photographs of OAP particles before and after white oil adsorption are displayed in Figure 9. An explicit distinction was observed between original OAP and swollen OAP. As shown in Figure 9a, before oil adsorption, a large amount of spherical particles with some cavities were accumulated together and formed an irregular surface, whereas, after reaching saturated adsorption, the small particles expanded significantly and filled in the gap space between the adjacent particles, forming a smooth surface.

### 3.3. Influence of OAP on the Properties of OBDFs

**Figure 10:**
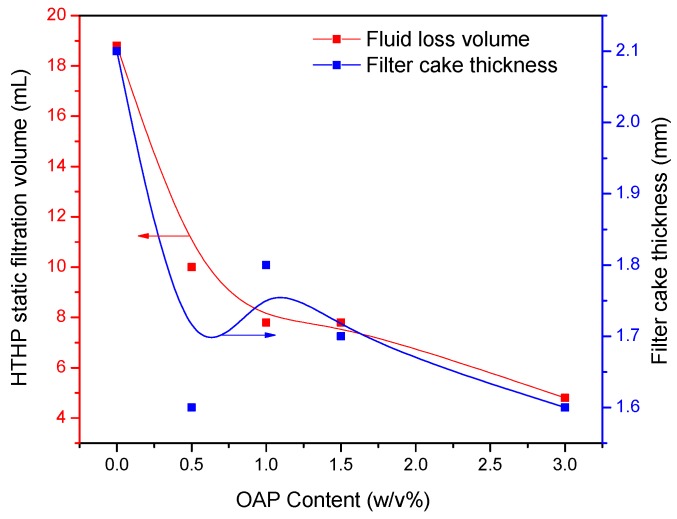
Variation of HTHP static filtration volume and filter cake thickness as a function of OAP content.

**Figure 11:**
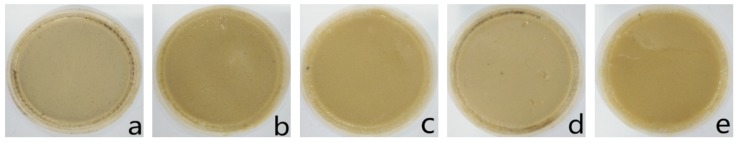
Photograph of HTHP filtration cakes with varying OAP contents. Note: (**a**) control sample; (**b**) 0.5 *w*/*v* % OAP; (**c**) 1.0 *w*/*v* % OAP; (**d**) 1.5 *w*/*v* % OAP; (**e**) 3.0 *w*/*v* % OAP.

The effect of OAP particles (particle size of 80–100 mesh) on the properties of OBDFs was investigated firstly. OAP with various contents was added into the OBDFs. The fluid was hot-rolled at 120 °C for 16 h. The properties of the fluids before and after hot rolling are given in Table 2. Before hot rolling, the addition of OAP had little influence on the rheological properties of the OBDFs, scuh that AV (calculated with Equation (2)), PV (calculated with Equation (3)), YP (calculated with Equation (4)) and Gel changed slightly with the increase of OAP content; however, after hot rolling, the rheological parameters including AV, PV, YP and Gel strength all increased gradually initially when the OAP content was lower than 1.5 *w*/*v* %, and showed a significant increase at content of 3 *w*/*v* %. In terms of filtration control, as shown in Table 2, the API fluid loss decreased gradually with the increasing content of OAP for both before and after hot rolling. Also as depicted in Figure 10 and Figure 11, for the HTHP static filtration test conducted under 120 °C and 3.5 MPa after hot rolling, both of the HTHP static fluid loss volume and filter cake thickness decreased with the increased content of OAP, which was reduced by 74% and 24%, respectively, when 3 *w/v* % OAP was added, indicating that OAP could effectively reduce the filtration loss and improve the filter cake quality. In regard to the electrical stability (ES) of the drilling fluid, which is one of the vital properties of an oil-based drilling fluid, this shows the voltage of the current flowing in the fluid and represents the emulsion stability of the fluid. The emulsion-breaking voltage decreased generally with the increasing content of OAP.

The impact of OAP particles on the properties of OBDF could be explained by the fact that, when OAP particles were added into the fluid initially, it began to adsorb oil, but the adsorptive content was relatively low. After hot rolling at high temperatures for a certain time, a large amount of oil was adsorbed by OAP, resulting in the decreased amount of free oil in the system. Meanwhile the swelled volume of OAP particles increased the friction of the fluid. The decreased content of free base oil and increased friction in the fluid contributed to the viscosity buildup, lower fluid loss and reduction of emulsion stability. Furthermore, the expansive OAP particles participated in forming the filter cake, which was also favorable for reducing filtration.

### 3.4. Sealing Properties of OAP

#### 3.4.1. High-Temperature and High-Pressure Filtration Test

**Figure 12:**
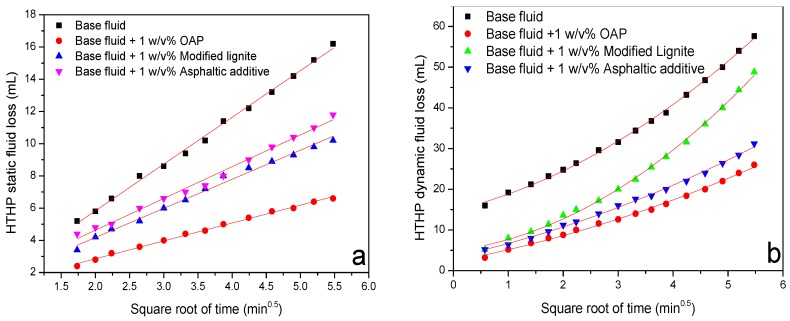
HTHP static fluid loss (**a**) and dynamic fluid loss (**b**) versus square root of time.

Two commercially available fluid loss additives including asphaltic additive and modified lignite were used as reference to compare the versatility of OAP. The fluid loss additives with 1 *w*/*v* % contents were added into the base formula of the OBDF and hot rolled at 120 °C for 16 h, and then the HTHP filtration tests were performed. As shown in Figure 12, for the HTHP static filtration test, the fluid loss volume was 32.4, 13.2, 20.4 and 23.6 mL for the base fluid, fluid containing OAP, modified lignite and asphaltic additives, respectively, showing the capacity of decreasing HTHP static filtration with the sequence of OAP > modified lignite > asphaltic additives. For the HTHP dynamic filtration test, the fluid loss volume increased to 115.2, 52.0, 97.6 and 62.4 mL for the four fluids, showing the ability of decreasing HTHP dynamic filtration following the order of OAP > asphaltic additive > modified lignite. The significant increase of fluid loss compared with that of static filtration lies in the fact that filter cake was more difficult to form under a dynamic shear condition [49]. Overall, the three candidate additives all exhibited effective performance in HTHP filtration control. 

The cumulative fluid loss volume of both HTHP static filtration test and HTHP dynamic filtration test as a function of square root of time was analyzed with different mathematic models. For HTHP static filtration, a typical model recommended by API standard was given as follows [50],

(5) $$\;V=V_{\mathrm{sp}}+m\sqrt{t}\;$$

where V_sp_ and m are the intercept and slope of the line, representing the spurt loss and filtration rate, respectively.

While for HTHP dynamic filtration test, another model describing the filtration in a dynamic test consisting of three separate steps: (1) spurt loss, (2) filter-cake deposition (square-root-of-time dependence), and (3) limitation of cake buildup by erosion (linear time dependence), was proposed by Roodhart [51] as follows,

(6) $$\;V=V_{\mathrm{sp}}+m\sqrt{t}+\mathrm{Bt}\;$$

where V_sp_ indicates the spurt loss, the constants m and B represent the static and dynamic filtration components. 

According to the proposed models (Equations (5) and (6)), the HTHP filtration test data were fitted and given in Table 3. It could be seen that the two models appeared to fit well as the value of R^2^ all approached to 1. With regard to the HTHP static filtration test, the addition of fluid loss reducers caused the increase of spurt loss, but lowered the filtration rates. The fluid in the presence of OAP generated the lowest filtration rate. For the HTHP dynamic filtration test, all the filtration components including spurt loss, static filtrate rate, and dynamic filtration rate decreased obviously after addition of the fluid loss reducers. The difference of filtrate rate between static and dynamic filtration was not clear, whereas, it was obvious that OAP was capable of reducing HTHP fluid loss more effectively than modified lignite and asphaltic additive under both static and dynamic conditions.

#### 3.4.2. Permeability Plugging Test

**Figure 13:**
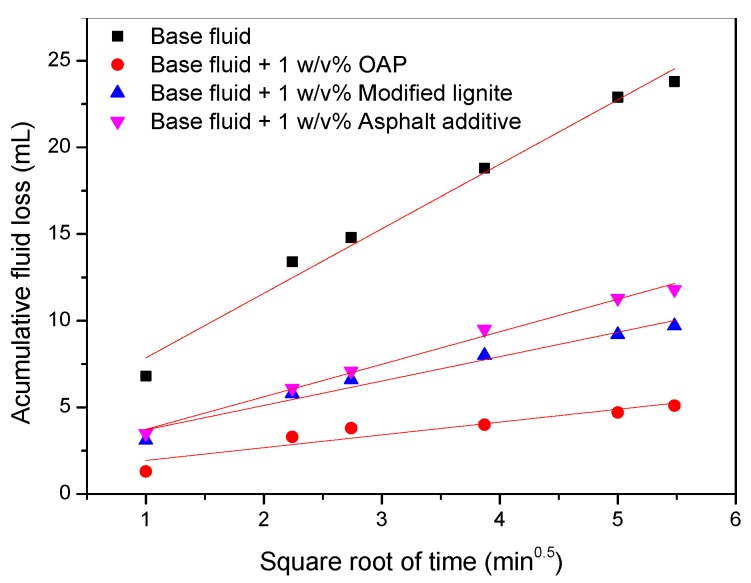
Fluid losses versus the square root of minutes for the permeability plugging test.

The results of the permeability plugging tests of OBDFs with and without fluid loss reducers are shown in Figure 13 and Table 4. The total loss of control sample was as high as 47.6 mL, indicating that base fluid was not able to plug the micro-pores of disks. However, an obvious decrease of total loss was observed after the addition of 1 *w*/*v* % fluid loss reducers. Furthermore, the static filtration rate decreased from 6.57 mL/min^1/2^ to 0.95, 2.26, 3.43 mL/min^1/2^ after incorporation of OAP, modified lignite, and asphalt additive, respectively. Because of the elastic and deformable properties, OAP particles could be easily squeezed into the micro-pores. After entering the micro-pores, the particles continued to absorb oil and swell to saturation state. The enlarged volume could effectively fill in the micro-pores and lead to the decrease of fluid loss.

#### 3.4.3. Sand Bed Filtration Test

**Figure 14:**
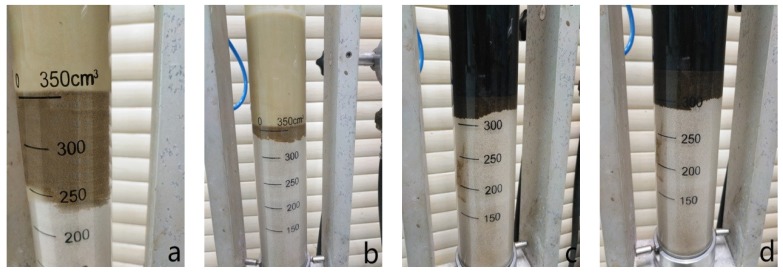
Comparison of the sand bed invasion depths for various fluid loss additives. Note: (**a**) control sample; (**b**) OAP; (**c**) asphaltic additive; (**d**) modified lignite.

The sand bed filtration test was designed to more realistically evaluate the tendency of a fluid to invade a permeable and unconsolidated formation [42]. As shown in Figure 14 and Table 5, when there was no sealing agent in the control sample, the base fluid invaded the sand bed readily and rapidly, and the total invasion depth reached about 7.2 cm after the test. After addition of sealing agents, the invasion depth decreased to a different extent. Compared with modified lignite and asphalt additive, OAP exhibited a better sealing performance. In the testing process, there was a small amount of initial invasion, followed by the rapid formation of a dense and extremely low permeability seal in just a few seconds. The fluid invasion ceased with an invasion depth of only 1.5 cm, which was the shortest invasion depth observed in contrast with other two products. This rapid shutoff of invasion was crucial in achieving the excellent results seen in the field. 

#### 3.4.4. Fracture Sealing Test

(1) The sealing capacity of OAP in OBDF with a 0.2 mm width slot.

**Figure 15:**
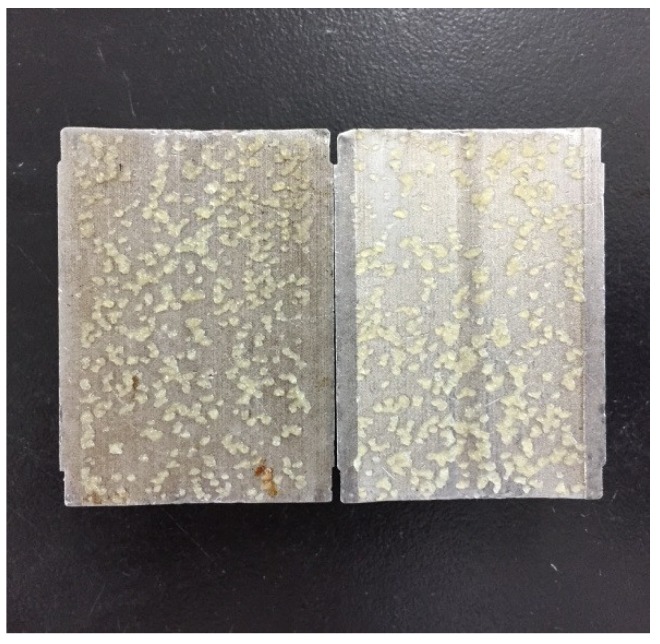
Performance of OAP to control the lost circulation in OBDFs (0.2 mm slot).

Firstly, the fracture sealing ability of OAP was evaluated by a 0.2 mm width slot to simulate light losses. OBDF with OWR of 80:20 containing 1 *w*/*v* % OAP with size of 80–100 mesh was poured into the cell. When the pressure increased to 1.0 MPa, almost all of fluid was lost, indicating sealing failure. After the experiment, the fracture was taken out. As shown in Figure 15, the swollen OAP particles had been forced into the fracture and were distributed along the fracture plane. Unlike rigid materials with high strength, OAP particles exhibited low compressive strength after oil adsorption, which could not form effective sealing alone. However, taking other materials such as SCC particles and sized RUB particles in combination, this relatively narrow fracture was easy to seal. 

(2) The sealing capacity of LCMs in a 1 × 0.5 mm width slot.

This fracture was selected to model light to medium losses. Because OAP individually could not effectively seal small fractures, to deal with this kind of loss it is widely accepted that combinations of LCMs are much more effective compared to just one type of LCM when curing lost circulation. The LCM’s type, concentration, particle size distribution and fracture width are the vital factors for effective sealing [52]. Based on general experience, rigid particles like calcium carbonate, deformable particles like rubber, and fibers were used in combination to improve the sealing capacity in this study. The particle size classification of LCMs is presented in Table 6. According to effective bridging theory [53] and numerous laboratory experiments, an acceptable LCM formula No. 1 aimed for the 1 × 0.5 mm tapered slot was established as a control sample and shown in Table 7.

**Figure 16:**
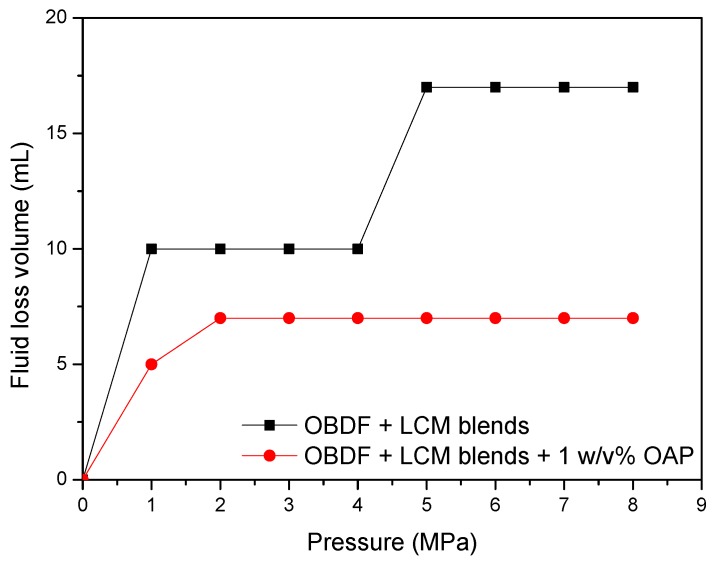
Performance of LCM blends and LCM blends combined with OAP to control the lost circulation in oil-based drilling fluids (1 × 0.5 mm slot).

**Figure 17:**
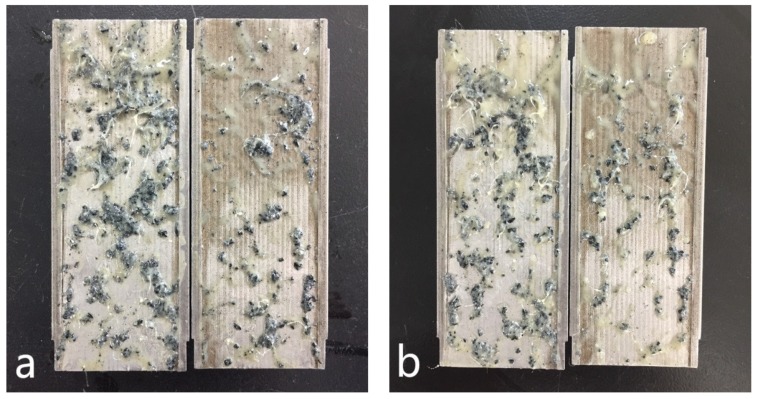
LCMs distribution in the slots after the fracture sealing test. Note: (**a**) formula No. 1; (**b**) formula No. 2.

The testing results shown in Figure 16 indicates that, for the control sample of formula No. 1, the fluid loss volume kept stable when the pressure less than 4.0 MPa, indicating the blended materials formed effective sealing in the fracture. After that, the fluid loss volume increased to 17 mL regardless of the increase of pressure. While for formula No. 2, the fluid loss volume was 5 mL at pressure of 1.0 MPa, then it increased to 7 mL and kept constant with the change of pressure, demonstrating that addition of OAP promoted formation of a tighter sealing in the fracture. As shown in Figure 17a, SCC particles and rubber particles were trapped with fibers to form a sealing integrity in the fracture. After OAP incorporation, as depicted in Figure 17b, SCC particles, rubber particles and OAP particles lodged against each other with the help of fibers binding. After rigid particles bridging, the resilient and deformable particles could fill in the formed voids, which was favorable for forming a tighter seal and lower fluid loss. 

(3) The sealing capacity of LCMs in a 2 × 1 mm width slot.

**Figure 18:**
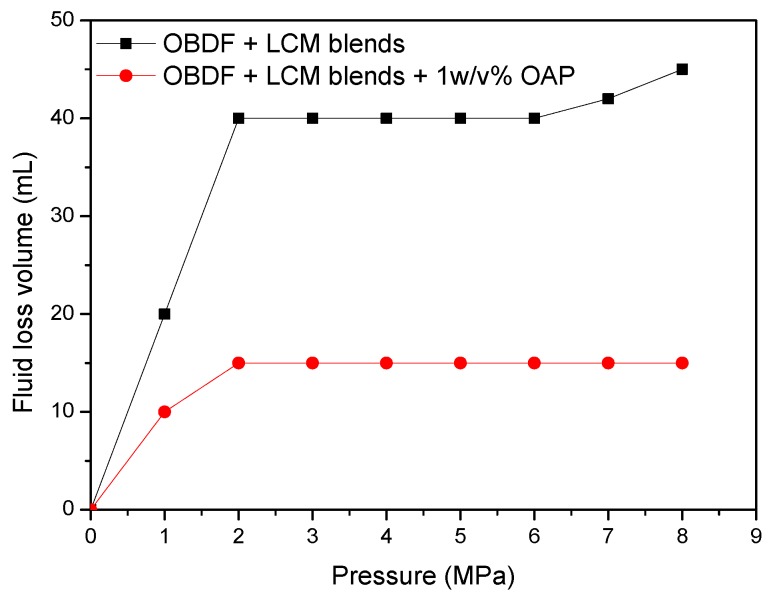
Performance of LCM blends and LCM blends combined with OAP to control the lost circulation in oil-based drilling fluids (2 × 1 mm slot).

**Figure 19:**
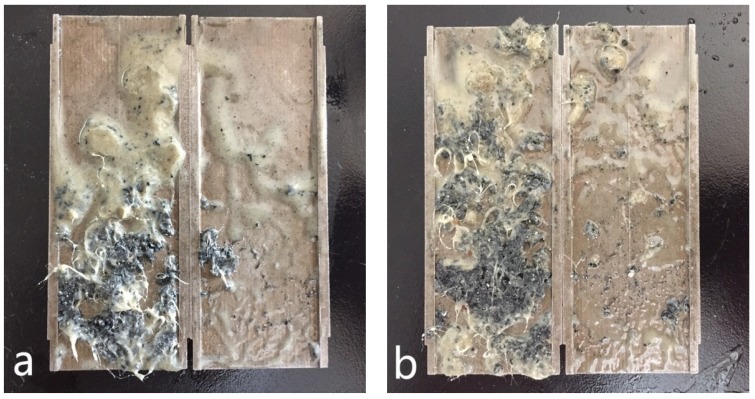
LCM distribution in the slots after the fracture sealing test. Note: (**a**) Formula No. 3, (**b**) Formula No. 4.

This type of slot was used to simulate moderate to severe losses. To form effective sealing, the SCC particles, rubber particles and fibers with appropriate size distribution and concentration were optimized after extensive experiments, then the optimized formula No. 3 was established as control sample and shown in Table 8. The fracture sealing test results for OBDFs containing formulas No. 3 and No. 4 are given in Figure 18. It was clear that, for both formulas No. 3 and No. 4, a jump of fluid loss was observed at pressure of 2.0 MPa. For formula No. 3, the fluid loss increased to 40 mL at 2.0 MPa and kept almost constant with the further increase of pressure. When the pressure reached 8.0 MPa, the fluid loss was 45 mL. A similar phenomenon was observed for formula No. 4, where the fluid loss increased to 15 mL and kept unchanged regardless of the increase of pressure, again indicating that incorporation of OAP contributed to a denser sealing and lower fluid loss. As shown in Figure 19a, it was seemed that the blended LCMs without OAP were mainly distributed on the tail of the fracture, which was relatively easy to be extruded out of the fracture and led to failure of sealing. While in the presence of OAP, as observed in Figure 19b, the blended LCMs filled along the entire fracture, beneficial to forming a steady sealing. The results also verified that the addition of OAP improved the retention of LCMs in the fractures, which in turn caused lowered fluid loss.

### 3.5. Probable Mechanism of Mitigation of Lost Circulation

OAP particles were synthesized by MMA, BA and LHA with typical crosslinked structure. The crosslinking chemicals tied the chains together to form a three-dimensional network, which enabled the polymers to absorb oil into the spaces in the molecular network and thus formed a gel that locks up the liquid. The oil-absorption process was an expansion process of this three-dimensional network, which had a clear transition from the high initial rate to the slow rate and toward the end of swelling equilibrium by the van der Waals attractive forces between molecules [54].

Because the oil absorption depended mainly on van der Waals attractive force, the oil absorption speed was relatively slow compared to that of water absorption resins; when pumped to downhole before absorption saturation, it would have a lower viscosity and small volume. Upon entering the lost-circulation zone, the absorption of oil continued, forming a viscous plug, and created a barrier to the subsequent flow of drilling fluid into the fracture channels. Meanwhile, after thorough adsorption under downhole conditions, the OAP particles swelled appreciably in volume and became resilient, which enabled them to be more effective in packing fractures even with smaller widths. However, due to their low compressive strength, the OAP particles were not efficient in closing off the lost circulation zone alone. When taking the rigid particles, elastic particles, fibers and OAP particles in combination by considering the particle shape, surface texture, concentration, particle size distribution and fracture width, a synergistic effect was obtained. The rigid SCC particles firstly formed bridging in the fracture, which was the framework of the sealing zone. The fibers that wrapped the mixture of other particles together improved the compactness of the sealing zone. After bridging in the fractures by rigid particles, deformable particles like OAP particles were able to occupy the voids among the previously bridged particles and reduce the permeability of the formed seal. Finally, a tight sealing zone was formed and resulted in a significant drop of fluid loss.

## 4. Conclusions

In this study, the oil-absorbent polymer (OAP) including MMA, SA, and BA was prepared by suspension polymerization. The OAPs had spherical and porous structure, and were capable of absorbing white oil and diesel oil several times of its own weight at temperatures ranging from room temperature to 120 °C. 

The addition of OAP had relatively little influence on the rheological properties of OBDF at content lower than 1.5 *w*/*v* % but increased the fluid viscosity remarkably at content higher than 3 *w*/*v* %. OAP particles could reduce the HTHP filtration and improve the sealing capacity of OBDFs effectively under downhole conditions. OAP particles used individually showed poor fracture-sealing capacity, but could effectively decrease fluid loss when treated conjunct with calcium carbonate particles, rubbers and fibers by a synergistic effect. 

After addition of OAP into the OBDF, the volume of OAP would increase with time, which resulted in the increase of viscosity of the fluid and slowed down the fluid loss speed. Meanwhile, the swelled and deformable OAP could be compressed to enter an opening that is substantially smaller and different in shape. OAP particles would conform to the openings with various shapes and sizes. More importantly, OAP particles could occupy the voids of the bridging layer, and resulted in a tight sealing zone when used in combination with other conventional LCMs.

## Figures and Tables

**Table 1:** Base formula of oil-based drilling fluids (OBDF) with oil to water ratio (OWR) of 90:10.

Component	Commercial Name	Content	Unit
White oil	No. 3	360	mL
Organic clay	VG-Plus	12	g
Primary emulsifier	BZ-OPE	12	g
Assistant emulsifier	BZ-OSE	12	g
Wetting agent	BZ-OWA	8	g
Rheological modifier	SD-RM	4	g
CaCl_2_ solution (25 wt %)		40	mL
CaO		8	g
Barite		As required	

**Table 2:** Effect of OAP on the properties of oil-based drilling fluid (OBDF) base formula.

Content (*w*/*v* %)	Testing Condition	AV (mPa·s)	PV (mPa·s)	YP (Pa)	Gel (Pa/Pa)	FL_API_ (mL)	ES (Volt)
0	BHR	11	9	1.9	1.5/2	7	2047
	AHR	12	9	2.9	2.0/3	7.4	
0.5	BHR	12.5	9	3.4	1.0/1.0	5.6	2047
	AHR	18	14	3.8	1.0/1.5	5.3	
1	BHR	15	12	2.9	1.0/1.5	4.8	1980
	AHR	28	18	9.6	1.5/2	3.1	
1.5	BHR	11.5	10	1.4	1.5/2	5.6	1562
	AHR	31	18	12.5	3.5/4.5	2.4	
3	BHR	17	10	6.7	1.5/1.75	2.2	1345
	AHR	98.5	65	32.2	8.0/9	1.2	

**Table 3:** HTHP static filtration and dynamic filtration test results for OBDFs containing various fluid loss additives.

Drilling Fluid Formula	HTHP Static Filtration			HTHP Dynamic Filtration			
	V_sp_	m	R^2^	V_sp_	m	B	R^2^
Base fluid	0.1075	2.8814	0.9982	14.771	3.2301	0.8218	0.9989
Base fluid + 1 *w*/*v* % OAP	0.4689	1.1507	0.9960	2.3177	2.5393	0.3072	0.9971
Base fluid + 1 *w*/*v* % Modified lignite	0.3295	1.8622	0.9942	4.9221	1.6198	1.1411	0.9984
Base fluid + 1 *w*/*v* % Asphaltic additive	0.7577	1.9535	0.9955	3.3494	2.9944	0.3557	0.9977

**Table 4:** Effect of various sealing agents on the fluid loss of OBDFs obtained via permeability plugging test.

Testing Sample	Total Fluid Loss (mL)	Spurt Loss (mL)	Static Filtration Rate (mL/min^1/2^)
Base fluid	47.6	11.6	6.57
Base fluid + 1 *w*/*v* % OAP	10.2	5	0.95
Base fluid + 1 *w*/*v* % Modified lignite	19.4	7	2.26
Base fluid + 1 *w*/*v* % Asphalt additive	23.6	4.8	3.43

**Table 5:** Comparison of invasion depths of various OBDF formulas for a sand bed filtration test.

Testing Sample	Average Invasion Depth (cm)
Base fluid	7.2
Base fluid + 1 *w*/*v* % OAP	1.5
Base fluid + 1 *w*/*v* % Modified lignite	2.2
Base fluid + 1 *w*/*v* % Asphaltic additive	2

**Table 6:** Classification of lost circulation materials’ (LCM) particle sizes.

Number	0	Ⅰ	Ⅱ	Ⅲ	Ⅳ
Mesh	6~10	10~20	20~40	40~80	>80
Particle size/mm	3.2~2.0	2.0~0.9	0.9~0.45	0.45~0.2	<0.2

**Table 7:** LCM formula for lost circulation control of oil-based drilling fluids in a 1 × 0.5 mm slot.

Testing Sample	SCC-Ⅱ	RUB-Ⅱ	SCC-Ⅲ	RUB-Ⅲ	SCC-Ⅳ	FIB	OAP
No. 1	2.0%	0.5%	3.0%	0.5%	1.0%	0.1%	-
No. 2	2.0%	0.5%	3.0%	0.5%	1.0%	0.1%	1.0%

**Table 8:** LCM formula for lost circulation control of oil-based drilling fluids in a 2 × 1 mm slot.

Testing Sample	SCC-I	SCC-Ⅱ	RUB-Ⅱ	SCC-Ⅲ	RUB-Ⅲ	SCC-Ⅳ	FIB	OAP
No. 3	4.0%	2.0%	0.5%	3.0%	0.5%	1.0%	0.2%	-
No. 4	4.0%	2.0%	0.5%	3.0%	0.5%	1.0%	0.2%	1.0%
